# Clonal hematopoiesis in patients with cancer and its association with risk of thrombosis and prognosis of disease

**DOI:** 10.1016/j.rpth.2025.102882

**Published:** 2025-05-08

**Authors:** Cornelia Englisch, Roland Jäger, Jasmina Gassner, Alice Assinger, Matthias Preusser, Peter Valent, Ingrid Pabinger, Cihan Ay

**Affiliations:** 1 Division of Hematology and Hemostaseology, Department of Medicine I, Medical University of Vienna, Vienna, Austria; 2 Department of Laboratory Medicine, Medical University of Vienna, Vienna, Austria; 3 Department of Vascular Biology and Thrombosis Research, Centre of Physiology and Pharmacology, Medical University of Vienna, Vienna, Austria; 4 Division of Oncology, Department of Medicine I, Medical University of Vienna, Vienna, Austria; 5 Ludwig Boltzmann Institute for Hematology and Oncology, Medical University of Vienna, Vienna, Austria

**Keywords:** cancer, clonal hematopoiesis, risk, thrombosis, venous thromboembolism

## Abstract

**Background:**

Patients with cancer are at high risk for cardiovascular events, especially venous and arterial thromboembolism (VTE/ATE). Clonal hematopoiesis (CH) has been identified as risk factor for cardiovascular diseases. However, there is limited insight into the impact of CH on thrombosis risk in patients with cancer.

**Objectives:**

The aim of this study was to elucidate the association between CH and cancer-associated VTE and ATE within the framework of the Vienna Cancer and Thrombosis Study (CATS), a prospective observational cohort study.

**Methods:**

Peripheral blood DNA samples collected at study inclusion were screened for CH-associated mutations.

**Results:**

In this study, 967 patients (median age: 61 [interquartile range, IQR: 50-68] years, 49.9% female) were included and followed-up for a median of 24 (IQR: 24-24) months. Of those, 787 (78.3%) had newly diagnosed cancer and 434 (44.9%) had stage IV disease. We identified 52 CH-associated variants in 46 patients (4.8%). Mutations in the genes *DNMT3A* (48.1%) and *TP53* (17.3%) were most commonly found. The presence of CH was not associated with VTE (adjusted subdistribution hazard ratio: 0.68, 95% CI: 0.21-2.19) or ATE risk (adjusted subdistribution hazard ratio: 1.08, 95% CI: 0.15-8.06). Available laboratory parameters and inflammatory and hemostatic biomarkers did not differ according to CH carrier status. Compared with patients without CH, those with CH showed decreased overall survival; however, this was not independent of age.

**Conclusion:**

In our cohort of patients with cancer, the presence of CH was not associated with an increased risk of VTE or ATE. CH had no independent impact on overall survival.

## Essentials

• Clonal hematopoiesis (CH) increases the risk of cardiovascular events and all-cause mortality.
• The influence of CH on venous and arterial thromboembolism (VTE/ATE) and mortality risk was assessed in patients with cancer.
• CH was not associated with risk of VTE or ATE in our cancer patient cohort.
• CH was not independently age-associated with all-cause mortality risk in our cancer cohort.

## 1 Introduction

Patients with cancer are at high risk for thrombotic complications such as venous thromboembolism (VTE) and arterial thromboembolic events (ATE). VTE and ATE risk in patients with cancer is about 7- to 9-fold and 2- to 3-fold higher, respectively, than that of the general population [1,2]. These complications often lead to treatment interruptions and delays and increased morbidity and mortality [[3], [4], [5], [6]].

Although various risk factors for cancer-associated VTE have been identified, the exact underlying mechanisms have not been fully elucidated. Based on the current knowledge, a substantial number of thromboembolic events in cancer patients cannot be explained, and even with existing tools for individual risk assessment in the clinical setting, >40% of patients who will develop a thromboembolic events are not captured as being high risk [7]. Thus, the implementation of risk assessment models and effective preventive strategies is significantly hampered in clinical practice. Therefore, more knowledge on risk factors for cancer-associated VTE and ATE is needed to identify patients at high risk more accurately.

Clonal hematopoiesis (CH) is defined as clonal expansion of a subset of hematopoietic stem cells due to an acquired somatic mutation—most commonly in the genes *DNMT3A*, *TET2*, or *ASXL1*—providing them with a survival benefit or proliferative advantage. CH has been noted to be prevalent in the general population and increasingly prevalent with age [[8], [9], [10]]. Interestingly, CH is associated with a high all-cause mortality risk that cannot solely be explained by the progression to hematological cancer [8]. It has been reported that CH is associated with cardiovascular diseases such as coronary heart disease, myocardial infarction, peripheral artery disease, and stroke [[11], [12], [13]]. Furthermore, it has been reported that CH is highly prevalent in patients with solid tumors, and DNA damage repair genes (eg, *TP53*, *PPM1D*) that cause CH are more frequently mutated [14,15].

The proposed mechanism of increased cardiovascular disease risk has been more closely examined in relation to certain common CH-associated mutations, such as *TET2* and *JAK2*. Enhanced inflammatory responses in monocytic, granulocytic, and lymphocytic cells have been suggested as contributing factors to the development of cardiovascular disease [11,16,17]. Additionally, activation of platelets and endothelial cells, which leads to increased P-selectin expression, has been proposed as a potential mechanism [16]. Given that platelet and endothelial cell activation, innate immune cells, and inflammation also play significant roles in venous thrombus formation [18], a potential association with venous thrombosis can be hypothesized. Only recently, CH, especially *JAK2*-driven CH, has been suggested to be associated with increased risk of VTE in the general population [19]. However, whether CH also contributes to the risk of VTE or ATE in patients with cancer requires further investigation.

The aim of our study was to assess the frequency of CH in a prospective cohort of cancer patients and its association with the risk of VTE, ATE, and all-cause mortality. Additionally, we sought to evaluate whether CH is associated with alterations in hemostatic biomarkers indicative of a hypercoagulable state in cancer patients.

## 2 Methods

### 2.1 Patient cohort

This project was performed within the framework of the Vienna Cancer and Thrombosis Study (CATS). This single-center, prospective observational cohort study recruited patients with newly diagnosed or recurrent cancer after full or partial remission for investigation of risk and risk factors for cancer-associated VTE between 2003 and 2019. The study was approved by the local ethics committee (EC number: 126/2003, ethik-kom@meduniwien.ac.at) and performed according to the Declaration of Helsinki and its later amendments. Detailed information about the study, its inclusion and exclusion criteria, and the study procedures have been described previously [20]. In brief, patients with histologically confirmed cancer and ≥18 years old were asked for written informed consent for study participation. Exclusion criteria included chemotherapy or VTE in the last 3 months, indication for long-term anticoagulation, and overt viral or bacterial infection, radiotherapy, or surgery 2 weeks prior to study inclusion. Additionally, patients with myeloid neoplasms were excluded from this analysis. For this project, 1021 patients with available whole-blood DNA samples were included, of which 54 had to be excluded from analysis due to insufficient and inconclusive total read coverage in the targeted resequencing analysis.

### 2.2 Outcomes of interest

Patients were followed for a maximum of 2 years for the primary outcome of interest, VTE, which includes symptomatic or fatal deep vein thrombosis, pulmonary embolism, and visceral vein thrombosis. Asymptomatic events were counted if the adjudication committee decided that it was of clinical significance and requested continuous anticoagulant treatment. Key secondary outcomes were ATE and all-cause mortality. All VTE and ATE events were adjudicated by an independent committee including experts in the fields of radiology, angiology (vascular medicine), dermatology, cardiology, neurology, or nuclear medicine.

### 2.3 Sample preparation

DNA was isolated from patients’ peripheral blood samples using the Maxwell RSC Blood DNA Kit on a Maxwell RSC Instrument (Promega). Routine parameters including hemoglobin, platelet and leucocyte counts, red cell distribution width (RDW), C-reactive protein (CRP), fibrinogen, factor (F)VIII activity, and D-dimer were measured centrally at the Department of Laboratory Medicine of the Medical University of Vienna. Soluble P-selectin (sP-selectin) was measured using a human sP-selectin immunoassay (R&D Systems) following the manufacturer’s instructions as described previously [20]. Extravesicular tissue factor activity were measured according to standardized protocols using a chromogenic endpoint assay, as reported in detail previously [21].

### 2.4 Targeted resequencing

Library preparation for targeted resequencing of selected genomic regions including hotspots for CH-associated mutations was performed as previously described [22] and expanded to include screening of selected DNA damage repair genes [23]. Therefore, genomic regions of interest involving the genes *DNMT3A*, *TET2*, *ASXL1*, *JAK2*, *PPM1D*, *CHEK2*, and *TP53* were amplified using multiplex polymerase chain reaction. Individual patient samples were subsequently indexed using the Nextera XT Index Kit v2 (Illumina) and purified using solid phase reversible immobilization with Agencourt AmPure XP magnetic beads (Beckman Coulter). Finally, library pools of up to 384 uniquely tagged samples were sequenced on a Illumina MiSeq instrument (MiSeq Reagent Kit v3 in 2 × 300 bp paired-end reads). Demultiplexing and subsequent somatic variant calling were performed using the Local Run Manager v2 software of the MiSeq instrument. Briefly, reads were aligned to the human reference genome version hg19 using the Burrows–Wheeler Aligner implementation of the Local Run Manager. A custom manifest file containing the amplicon regions of interest was used. Somatic variants were called from a variant allele frequency of 0.01 at a minimum variant site depth of 10. Indel Realignment was switched on. All patient samples included in the study underwent targeted resequencing in a screening run. Samples carrying positively selected variants were subjected to a validation run, which involved a second round of sequencing subsequent to a separate library preparation under identical conditions.

### 2.5 Variant annotation and filtering

For variant annotation, RefSeq was selected. Variants were filtered as previously described [22]. In brief, the following inclusion criteria were used: variant site depth ≥100, variant reported as somatic in Catalogue of somatic mutations in cancer (COSMIC) [24], population allele frequency <0.1% in Genome aggregation database (gnomAD) databases [25], protein-coding, nonsynonymous, variant allele frequency (VAF) between 2% to 40% or 60% to 90% (except for *JAK2*^*V617F*^ mutations, for which all VAF ≥2% was accepted). For variants passing filters applied on the screening run, samples were rerun to confirm validity at adjusted filter settings (inclusion criteria for validation run: variant site depth ≥200, VAF ≥1%). While variants not passing the validation criteria were removed, variant call characteristics including VAFs for validated variants were exported from the screening run.

### 2.6 Statistical considerations

All statistical analysis were performed with IBM SPSS (version 28.0) and R Studio (version 4.2.3). Standard statistical summary modes were used to depict patient characteristics (absolute and relative frequencies, median and interquartile range [IQR]). The α-level was set at 0.05. Follow-up time was calculated using the reverse Kaplan–Meier method. A binary sex categorization (male/female) as designated at birth was used.

Due to the high mortality rate in our cohort, VTE and ATE outcomes were assessed in a competing risk framework with all-cause mortality as competing event of interest. Cumulative incidence plots were generated with competing risk functions. A proportional Fine and Gray subhazard regression model was conducted to compare between-group differences. All-cause mortality was assessed with Kaplan–Meier analysis and compared using a log-rank test. Furthermore, Cox regression analysis was performed to assess the association between CH and all-cause mortality. Multivariable analyses included age, sex, cancer type, and stage. Differences of clinical characteristics and laboratory parameters or biomarkers of interest were assessed with a chi-squared test or a Mann–Whitney U-test, as appropriate. Previous radiotherapy was defined as any exposure to therapeutic radiation ≥2 weeks before study inclusion, while previous chemotherapy was defined as the administration of any cytotoxic agent for cancer treatment ≥3 months prior to study inclusion. A subgroup analysis was performed on the group of patients with tumor types considered to have a low thrombotic risk, including breast and prostate cancers [26].

## 3 Results

### 3.1 Patient characteristics

The total study cohort in this analysis consisted of 967 patients with cancer. The median age was 61 years (IQR: 50-68) and of all patients, 49.9% were female. Of patients, 787 (78.3%) had newly diagnosed cancer at study inclusion. The 3 most common tumor types were lung (21.0%), breast (16.4%), and brain (10.2%) cancer, and 434 (44.9%) had stage IV disease at study inclusion. Detailed patient characteristics are presented in Table 1.

**Table 1:** Characteristics of patients (n = 967) at study inclusion.

Characteristics	Patients with available data (n)	*n* (%) or median (IQR)
Female	967	483 (49.9)
Age (y)	967	61 (50-68)
Body mass index (kg/m^2^)	962	24.9 (22.3-28.1)
Median follow-up time (mo)	967	24 (23-24)
Newly diagnosed	967	757 (78.3)
Stage IV (vs I, II, III)	966	434 (44.9)
Tumor type	967	
Brain		184 (19.0)
Breast		159 (16.4)
Lung		203 (21.0)
Stomach		15 (1.6)
Colorectal		73 (7.5)
Pancreas		73 (7.5)
Kidney		9 (0.9)
Prostate		38 (3.9)
Multiple myeloma		20 (2.1)
Lymphoma		99 (10.2)
Other		94 (9.7)
Current or prior smoking exposure	745	376 (38.9)
Comorbidities	967 (0)	
Arterial hypertension		351 (36.3)
Diabetes mellitus		102 (10.5)
Hyperlipidemia		95 (9.8)

### 3.2 CH mutations

**Figure 1:**
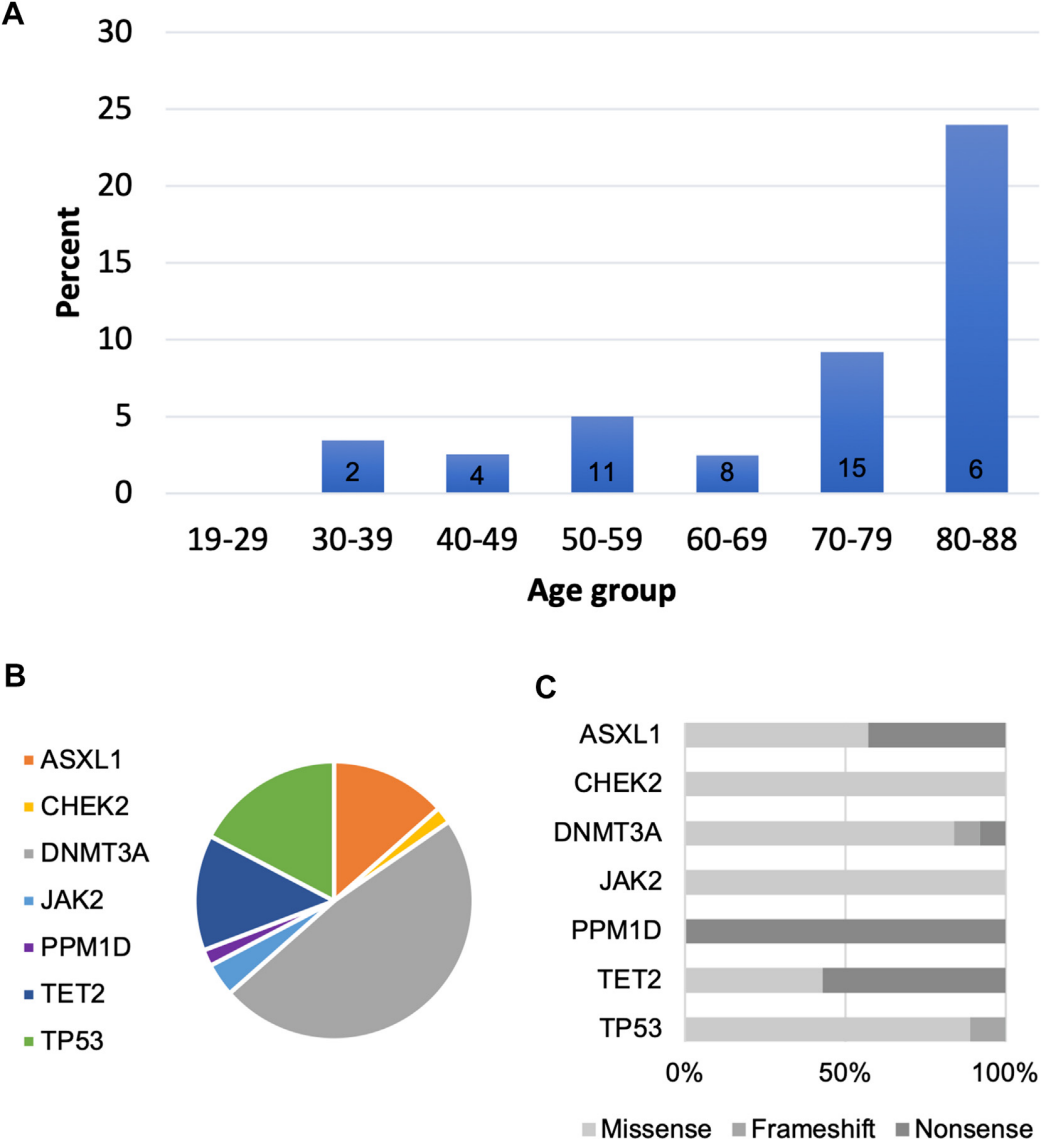
Characteristics of the clonal hematopoiesis (CH) mutations detected in the Vienna Cancer and Thrombosis Study. (A) Percentage of patients with CH carrier status stratified according to age group. Numbers in the bars indicate absolute numbers of patients with CH-associated mutation. (B) Frequency of genes found with mutation. (C) Types of mutations detected according to gene.

Overall, we detected 52 CH-associated variants in 46 (4.8%) patients, and the frequency increased with age. The mutations occurred most frequently in *DNMT3A* (48.1%) and *TP53* (17.3%). The 52 variants had a median VAF of 4.85 (IQR: 3.05-12.82) and were most commonly single nucleotide variants (94.2%) and missense (75%) mutations (Figure 1).

Patients with CH were significantly older than patients without CH (median age 67 vs 60 years, *P* = .002). Among 46 patients with CH, 22 (47.8%) were female, which was comparable with the gender distribution in the whole cohort. Patients with CH were equally likely to be current smokers or have a history of smoking exposure (30.4% vs 39.3%, *P* = .59). No patient with an *ASXL1* mutation had current or past smoking exposure. Furthermore, individuals with CH were equally likely to have newly diagnosed cancer (80.4% vs 78.2%, *P* = .86) and to have a history of prior chemo- or radiotherapy (8.7% vs 9.3% and 8.7% vs 10.5%, respectively). Two patients with a history of chemo- and radiotherapy had a *DNMT3A* mutation, 1 patient each with a history of radiotherapy had a *TP53* and *DNMT3A* mutation, and 1 patient each with previous chemotherapy exposure had a *TP53* and *DNMT3A* mutation. Most mutations were found in patients with brain (*n* = 13, 28.3%), lung (*n* = 9, 19.6%), and prostate cancer (*n* = 4, 8.7%) (Supplementary Table S1). When investigating the subgroup of patients with DNA damage repair (DDR) gene (*CHEK2*, *PPM1D*, *TP53*) mutation, their characteristics did not differ significantly compared with the rest of patients, but those with DDR gene mutations again tended to be older (Supplementary Table S2).

### 3.3 Association of CH with VTE and ATE

Over a median follow-up period of 24 months (IQR: 24-24), 86 VTE and 18 ATE events occurred. This translates into a 6-, 12-, 24-month cumulative VTE incidence of 5.8% (95% CI: 4.3-7.4), 7.7% (95% CI: 6.0-9.4), 9.4% (95% CI: 7.5-11.3) and a 6-, 12-, 24-month cumulative ATE incidence of 0.8% (95% CI: 0.2-1.4), 1.5% (95% CI: 0.7-2.3), and 2.0% (95% CI: 1.0-2.9), respectively.

**Figure 2:**
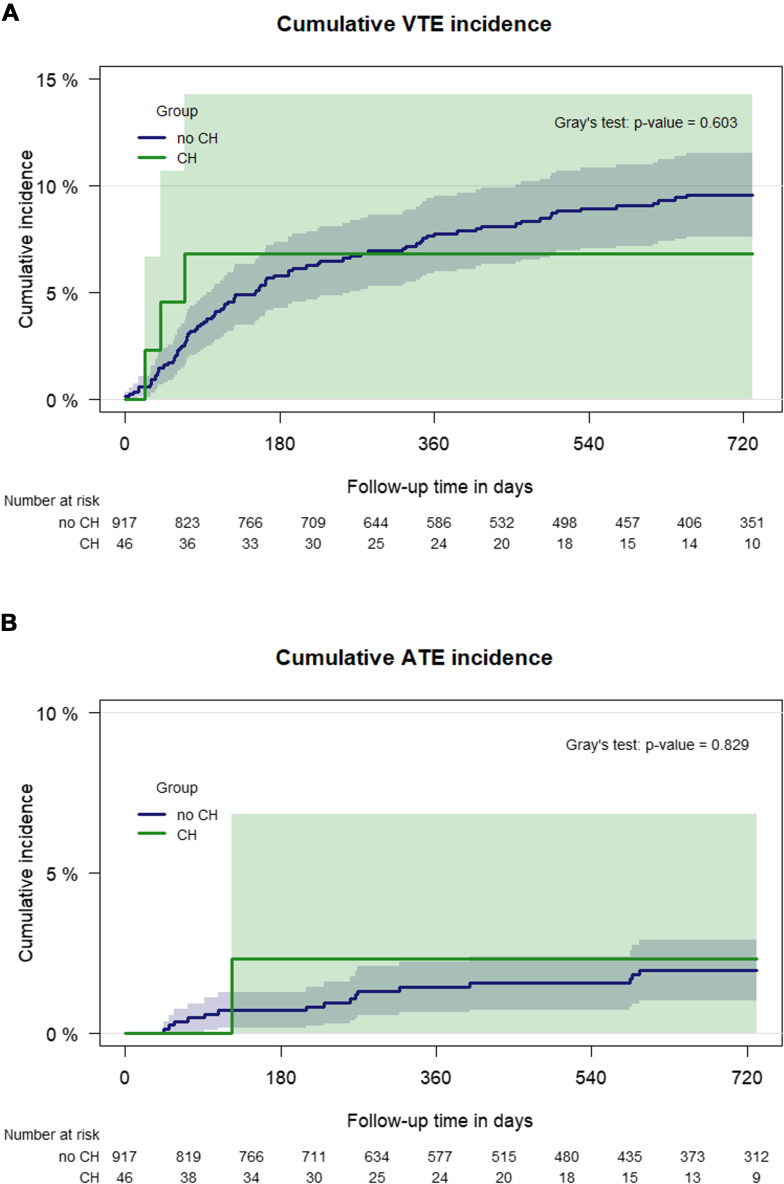
Cumulative (A) venous thromboembolism (VTE) and (B) arterial thromboembolism (ATE) incidence in patients with and without clonal hematopoiesis (CH) carrier status. Patients were divided into 2 groups according to the CH carrier status, and the 2 groups were compared within a Fine and Gray subdistribution hazard model.

Of 46 patients with CH, 3 (6.5%) had a VTE event during the observation period, whereas 89 of 921 patients (9.6%) without CH had a VTE event. Thus, patients with CH (*n* = 46) had a similar 12-month cumulative VTE incidence as those without CH (*n* = 921): 6.8% (95% CI: 0.0-14.3) versus 7.8% (95% CI: 6.0-9.5; Gray’s test *P* = .60; Figure 2A). The presence of CH was neither associated with an increased risk of VTE in univariable (subdistribution hazard ratio [SHR]: 0.74, 95% CI: 0.23-2.38) nor in multivariable analysis (adjusted SHR: 0.68, 95% CI: 0.21-2.19). Of the patients with CH and VTE, 2 had a mutation in *TP53* and 1 in *ASXL1*.

Among 46 patients with CH, 1 (2.2%) experienced an ATE event during the observation period, compared to 15 of 921 patients (1.6%) without CH. The 12-month cumulative incidence of ATE was similar between patients with CH (2.3%, 95% CI: 0.0-6.8) and those without CH (1.4%, 95% CI: 0.6-2.2; Gray’s test *P* = .83, Figure 2B). The presence of CH was not associated with an increased risk of ATE in either univariable analysis (SHR: 1.25, 95% CI: 0.17-9.43) or multivariable analysis (adjusted SHR: 1.08, 95% CI: 0.15-8.06). The patient with CH who developed ATE had a mutation in the *DNMT3A* gene.

Further subgroup analyses of CH and VTE/ATE stratified according to cancer type are summarized in Supplementary Table S3 and S4.

### 3.4 Association of CH with all-cause mortality

During the observation period, 442 patients died, which translates to a 12- and 24-month overall survival of 72.7% (95% CI: 69.9-75.6) and of 54.3% (95% CI: 51.1-57.7), respectively.

**Figure 3:**
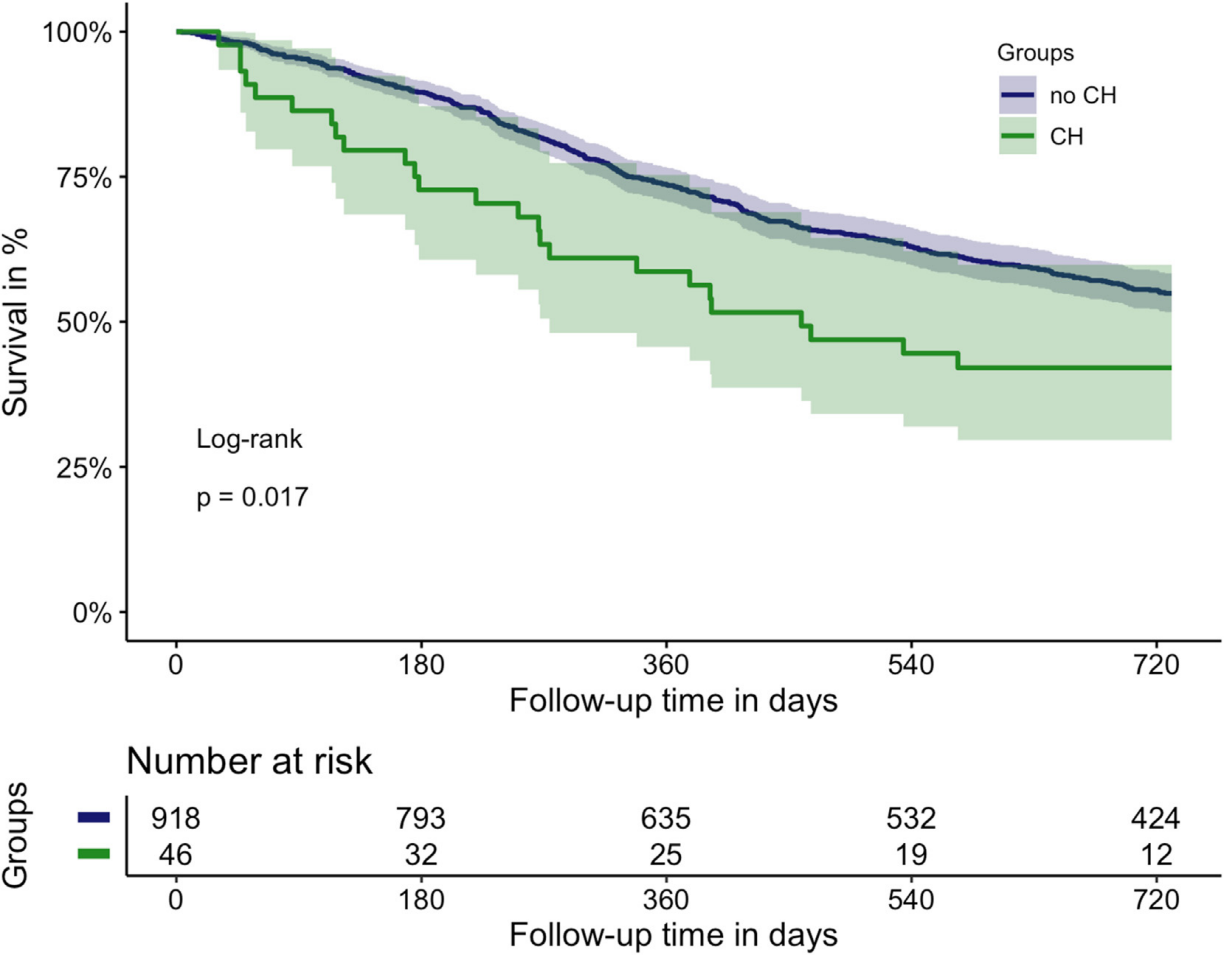
Overall survival in patients with (*n* = 46) and without (*n* = 921) clonal hematopoiesis (CH). Patients were divided according to their CH carrier status and the groups were compared within a Kaplan–Meier analysis and with a log-rank test.

Patients with CH had a poor overall survival compared with patients without CH (12-month survival: 58.7% [95% CI: 45.7-75.3] vs 73.4% [95% CI: 70.5-76.4], *P* = .02, Figure 3). CH was associated with all-cause mortality risk in univariable (hazard ratio [HR]: 1.63, 95% CI: 1.09-2.44), but not in multivariable analysis after adjustment for age, sex, cancer type, and stage (adjusted HR: 1.33, 95% CI: 0.89-2.00; Supplementary Figure S1). Causes of death were mostly cancer-related, and only 2 patients without CH exhibited a cardiovascular reason of death.

Furthermore, 11 patients with a CH-associated mutation in a DDR gene (*CHEK2*, *PPM1D*, *TP53*) had significantly worse overall survival than all other patients (12-month survival: 54.5% [95% CI: 31.8-93.6] vs 72.9% [95% CI: 70.1-75.8], *P* = .03; Supplementary Figure S2). Presence of a DDR gene mutation was also associated with increased all-cause mortality risk in univariable analysis (HR: 2.18, 95% CI: 1.08-4.40), but not in multivariable analysis (adjusted HR: 1.69, 95% CI: 0.84-3.42).

### 3.5 Association between CH and laboratory parameters and biomarkers

The median level of laboratory parameters, including hemoglobin, leukocyte, platelet count, and red cell distribution width (RDW) did not differ between patients with and without CH (Table 2). Interestingly, patients with DDR gene mutation exhibited higher leukocyte counts and RDW than patients without a mutation in a DDR gene (Supplementary Table S3). CRP levels did not differ between individuals with and without CH (0.45 vs 0.41 mg/dL, *P* = .47; Table 2), and also not in those with DDR gene mutation.

**Table 2:** Laboratory values and hemostatic biomarker levels in all patients and in patients with and without clonal hematopoiesis.

Parameters	All (n = 967)	CH (*n* = 46)	No CH (*n* = 921)	*P*
Hemoglobin (g/dL)	13.1 (11.9-14.2)	13.0 (11.7-13.9)	13.0 (11.9-14.2)	.43
Platelets (×10^9^/L)	261 (207-321)	255 (195-327)	261 (209-320)	.55
Leucocytes (×10^9^/L)	7.63 (6.11-10.04)	7.84 (6.20-10.99)	7.64 (6.11-10.04)	.42
RDW (%)	13.8 (13.1-14.6)	14.0 (13.2-15.0)	13.8 (13.1-14.6)	.31
CRP (mg/dL)	0.42 (0.14-1.44)	0.45 (0.20-1.60)	0.42 (0.14-1.44)	.47
Fibrinogen (mg/dL)	376 (307-466)	381 (321-435)	376 (305-467)	.97
Factor VIII activity (%)	198 (153-251)	221 (164-269)	198 (153-250)	.13
D-dimer (μg/mL)	0.74 (0.39-1.59)	0.88 (0.58-1.61)	0.71 (0.39-1.57)	.09
sP-selectin (ng/mL)	34.30 (25.33-44.00)	32.35 (36.80-39.5)	34.60 (25.20-44.10)	.46
EV-TF (pg/mL)	0.17 (0.06-0.28)	0.09 (0.0-0.17)	0.18 (0.07-0.28)	.33

Data presented as median (IQR). Differences between columns were assessed with a Mann–Whitney U-test.

CH, clonal hematopoiesis; CRP, C-reactive protein, EV-TF, extravesicular tissue factor; RDW, red cell distribution width.

Also, hemostatic biomarkers including sP-selectin, fibrinogen, FVIII activity, and extravesicular tissue factor activity did not differ significantly between CH carriers and unaffected individuals (Table 2). D-dimer levels tended to be higher (0.88 vs 0.71 μg/mL, *P* = .09) in patients with CH. Patients with DDR gene mutation had higher FVIII activity levels than patients without a mutation in DDR genes (273% vs 198%, *P* = .02; Supplementary Table S3).

As cancer itself is known to be a driver of inflammation and activation of hemostasis [[27], [28], [29]], we evaluated the difference in CRP levels and hemostatic biomarkers in the subgroup of patients with tumor types considered to have a low thrombotic risk (breast and prostate cancer) [26]. This subgroup included 7 patients with CH and 190 patients without CH. There was no difference between 2 groups when evaluating CRP, sP-selectin, fibrinogen, FVIII activity, and D-dimer levels (Supplementary Table S4).

## 4 Discussion

In this study, we assessed the prevalence of CH-associated mutations in a well-characterized cohort of patients with various types of cancer. The main aim of our analysis was to investigate the role of CH in cancer-associated thrombosis (both VTE and ATE), and our results indicated no association. Of note, CH was linked to a poor overall survival in patients with cancer while the association was weakened after additional adjustment for age.

The frequency of CH in patients with cancer has been reported to be higher than in the general population [14,15]. However, in our cohort of patients with cancer, we observed a generally expected CH frequency, which might be attributable to the fact that about 80% of the included patients had newly diagnosed cancer. This hypothesis is supported by the fact that previous studies investigated cohorts including a larger proportion of patients with previous exposure to radiation and chemotherapy [15]. Additionally, a study assessing individuals affected with first-time primary cancer without prior radiation or chemotherapy found a low CH frequency of 2% [30]. Furthermore, the varying methodology and different CH definitions used hamper a comparison between studies [14,15,30]. Nevertheless, prior radiation and chemotherapy were proposed to be a cause for an increased CH frequency and to especially lead to a higher frequency of DDR gene mutations [14]. In our cohort, we did not find an association between CH, or more specifically, DDR gene mutations, and a history of prior chemotherapy or radiation exposure. It is worth noting that CH was originally studied in the context of myeloid neoplasms, and this research revealed that the latency period between treatments such as chemotherapy or radiation and the development of neoplasms is often several years. This may explain why we did not observe an elevated CH frequency in these patients. Moreover, we did not observe that patients with CH were more likely to have current or previous smoking exposure. This lack of association persisted even when specifically analyzing *ASXL1* mutations, which have been reported to be particularly linked to smoking exposure [14,[31], [32], [33]]. Interestingly, we found that patients with primary brain cancer exhibited the highest CH frequency, which warrants further research in this patient group.

CH has evolved as a major clinical and research topic in recent years. It has been reported that CH is prevalent in the general aging population and associated with increased all-cause mortality, independent of progression to hematological cancer [8]. Interestingly, although there is a clear association between (1) CH and age and (2) CH and myeloid neoplasms (which often also predispose to VTE and ATE), CH was discovered as a more or less independent cardiovascular risk factor and may increase the risk of coronary heart disease, myocardial infarction, stroke, and peripheral artery disease [8,[11], [12], [13],^34^]. The association with other cardiovascular diseases such as VTE, however, remains less well researched. While small cohort studies found a higher CH prevalence in patients with unprovoked VTE than would have been expected in the general population [35,36], there was no comparison to matched healthy individuals or closer evaluation of a potential causal relationship. A very recent UK Biobank study revealed an association between CH and VTE risk in the general population, most strongly when *JAK2* was mutated [19]. In contrast, in our cohort of patients with cancer, CH was not associated with increased VTE risk. So far, only one previous study investigated the role of CH with cancer-associated VTE and, similar to our study, found no association with increased risk of VTE [37]. Cancer is per se a main driver of VTE risk and development and therefore, risk factors of only mild to moderate magnitude can be overshadowed by the cancer itself; hence, the CH-driven risk might have been masked. This phenomenon was also previously observed in relation to factors that are clearly associated with an increased risk of VTE in the general population but have a less clear association in cancer patients, such as blood type [38]. We also did not observe an association between CH and ATE in our cohort. This finding has to be interpreted with caution as the wide confidence interval indicates high uncertainty of estimates. The power to detect a significant association was lower compared with our VTE analysis, as there were fewer ATE events. Because no previous studies have assessed whether CH is associated with ATE in the cancer population, future investigations in larger cohorts with a higher event rate are needed to close this current knowledge gap. Finally, our results must be interpreted considering the selected cohort, which included patients with solid tumors, lymphoma, and multiple myeloma but excluded those with myeloid malignancies, which themselves have a high CH frequency and are linked to thrombotic risk. Thus, this could be another reason for the lack of association between CH and VTE and ATE risk in our cohort, especially in patients with hematological malignancy. Furthermore, some patients with CH may have experienced thrombotic events and started anticoagulation before a formal cancer diagnosis, leading to their exclusion from our study and limiting insights into this group.

The proposed mechanism underlying the association between CH and cardiovascular disease involves an enhanced proinflammatory environment [39]. Pivotal mouse models elucidated that macrophages harboring CH mutations in particular contribute to the phenotype of increased plaque formation and elevated levels of proinflammatory cytokines [11,16]. Although the results were conflicting, a few previous studies reported that patients with CH exhibited higher CRP levels than those without CH mutations [[40], [41], [42]]. In our cohort study of patients with cancer, CRP levels did not differ according to the presence of a CH mutation. Moreover, we did not observe a difference in hemostatic biomarkers when stratified according to CH carrier status. Inflammation is one hallmark of cancer, and cancer also displays a tight interaction with the hemostatic system, leading to a prothrombotic state. It is important to point out that patients with cancer often present with increased levels of both inflammatory parameters (especially CRP), reflecting cancer-related inflammation, and hemostatic biomarkers (eg, fibrinogen, D-dimer, FVIII activity), indicating cancer-induced hypercoagulability [[27], [28], [29]]. Therefore, cancer itself may be the stronger trigger of inflammation and hypercoagulability, overwhelming the potential for CH-induced inflammation or hypercoagulability.

Poor overall survival in cancer patients with CH was previously reported, in particular in patients with solid tumors and lymphoma undergoing autologous stem cell transplantation [15,43]. We also observed worse overall survival in patients with CH in our cancer cohort, and interestingly, this was also seen in the smaller subgroup of patients with DDR gene mutation. While previous studies reported the association between survival and CH in cancer patients to be independent of age, gender, and smoking status [15], in our cohort, CH was not independently associated with survival after adjustment for age. Thus, while our cohort is representative of a cancer center population, a population-based approach appears necessary to analyze independent associations, particularly those independent of age. On the other hand, this may also indicate that cancer is a stronger driver that might trump the effect of other milder factors such as CH, in contrast to the general population in which such a strong shadowing factor is not present. As anticipated, most deaths were attributed to cancer progression, while 2 patients without CH died from cardiovascular causes, which was the rationale for not examining the impact of CH on cardiovascular mortality.

There are a few limitations that merit mentioning. First, our cohort was rather small, especially to assess a potential link between CH and ATE. Further research should combine large cohorts (with high ATE and VTE incidence) and detailed follow-up data. Second, the low number of detected CH variants limited our ability to study the specific mutated genes individually in detail. However, this is a cohort that was meticulously followed-up, for which close details and laboratory parameters were available, and all events were adjudicated by independent experts. Moreover, no interleukin levels (IL), such as IL-1β and IL-6, were available for analysis, and we could only use CRP levels to analyze a potential association between CH and inflammation in our cohort. Third, our analyses were restricted to mutational hotspots within selected genes, potentially resulting in an underestimation of CH frequencies. Nevertheless, the 7 genes included in our screening assay comprise the majority of mutated genes described in previous pivotal studies of CH [8,14].

In conclusion, we did not find an association between CH and cancer-associated VTE or ATE. Cancer itself is probably the strongest risk factor, thereby overshadowing other mild to moderate factors. The association between ATE and CH in patients with cancer needs future research in large cohort studies. However, CH was not independently associated with overall survival after adjustment for age, and further investigations are needed to reveal more insights.
