# Supplementary material for: Clonal hematopoiesis in patients with cancer and its association with risk of thrombosis and prognosis of disease
Source: Res Pract Thromb Haemost. 2025 May 8;9(4):102882. doi: 10.1016/j.rpth.2025.102882 (PMC12167797; doi:10.1016/j.rpth.2025.102882)
Supplement: Supplementary Material [file mmc1.docx]

**Supplement:
Clonal hematopoiesis in patients with cancer and its association with risk of thrombosis and prognosis of disease**

Cornelia Englisch^1^, Roland Jäger^2^, Jasmina Gassner^1,2^, Alice Assinger^3^, Matthias Preusser^4^, Peter Valent^1,5^, Ingrid Pabinger^1^, Cihan Ay^1^

^1^Division of Hematology and Hemostaseology, Department of Medicine I, Medical University of Vienna; Vienna, Austria

^2^Department of Laboratory Medicine, Medical University of Vienna; Vienna, Austria

^3^Department of Vascular Biology and Thrombosis Research, Centre of Physiology and Pharmacology, Medical University of Vienna; Vienna, Austria

^4^Division of Oncology, Department of Medicine I, Medical University of Vienna; Vienna, Austria

^5^Ludwig Boltzmann Institute for Hematology and Oncology, Medical University of Vienna; Vienna, Austria

**Table of content**

Supplementary table S13

Supplementary table S24

Supplementary table S35

Supplementary table S46

Supplementary table S57

Supplementary table S68

Supplementary figure S19

Supplementary figure S210

**Supplementary table S1. Patient characteristics at study inclusion of patients stratified according to their clonal hematopoiesis carrier status.**

|  | **CH (n=46)** | **No CH (n=921)** | **p value** |
| --- | --- | --- | --- |
| **Age** | 67 (55-72) | 60 (49-67) | 0.002 |
| **Female** | 22 (47.8) | 461 (50.1) | 0.88 |
| **BMI** | 25.5 (22.7-27.8) | 24.8 (22.2-28.1) | 0.63 |
| **Newly diagnosed** | 37 (80.4) | 720 (78.2) | 0.86 |
| **Stage 4 vs other** | 23 (50) | 411 (44.6) | 0.76 |
| **Tumor type** |  |  | 0.2 |
| **Brain** | 13 (28.3) | 171 (18.6) |  |
| **Breast** | 3 (6.5) | 156 (16.9) |  |
| **Lung** | 9 (19.6) | 194 (21.1) |  |
| **Stomach** | 0 | 15 (1.6) |  |
| **Colorectal** | 2 (4.3) | 71 (7.7) |  |
| **Pancreas** | 2 (4.3) | 71 (7.7) |  |
| **Kidney** | 0 | 9 (1) |  |
| **Prostate** | 4 (8.7) | 34 (3.7) |  |
| **Multiple Myeloma** | 1 (2.2) | 19 (2.1) |  |
| **Lymphoma** | 4 (8.7) | 95 (10.3) |  |
| **Other** | 8 (17.5) | 86 (9.3) |  |
| **Prior chemotherapy** | 4 (8.7) | 86 (9.3) | 0.88 |
| **Prior radiotherapy** | 4 (8.7) | 97 (10.5) | 0.67 |
| **Current or prior smoking exposure** | 14 (30.4) | 362 (39.3) | 0.59 |

Values are presented as absolute numbers (percentages) or median (interquartile range). Percentages are calculated per column. Differences between columns were assessed with a chi-square test or a Mann-Whitney U test as appropriate. CH – clonal hematopoiesis

**Supplementary table S2. Patient characteristics at study inclusion of patients stratified according to their DNA damage repair gene mutation carrier status.**

|  | **DDR gene mutation (n=11)** | **No DDR gene mutation  (n=956)** | **p value** |
| --- | --- | --- | --- |
| **Age** | 67 (56-70) | 61 (50-68) | 0.09 |
| **Female** | 4 (36.4) | 479 (50.1) | 0.55 |
| **BMI** | 27.4 (23.3-33.0) | 24.9 (22.2-28.1) | 0.17 |
| **Newly diagnosed** | 8 (72.7) | 749 (78.3) | 0.71 |
| **Stage 4 vs other** | 7 (63.6) | 427 (44.7) | 0.45 |
| **Tumor type** |  |  | 0.44 |
| **Brain** | 3 (27.3) | 181 (18.9) |  |
| **Breast** | 0 | 159 (16.6) |  |
| **Lung** | 1 (9.1) | 202 (21.1) |  |
| **Stomach** | 0 | 15 (1.6) |  |
| **Colorectal** | 1 (9.1) | 72 (7.5) |  |
| **Pancreas** | 1 (9.1) | 72 (7.5) |  |
| **Kidney** | 0 | 9 (0.9) |  |
| **Prostate** | 2 (18.2) | 36 (3.8) |  |
| **Multiple Myeloma** | 0 | 20 (2.1) |  |
| **Lymphoma** | 2 (18.2) | 97 (10.1) |  |
| **Other** | 1 (9.1) | 93 (9.7) |  |
| **Prior chemotherapy** | 2 (18.2) | 88 (9.2) | 0.27 |
| **Prior radiotherapy** | 1 (9.1) | 100 (10.5) | 0.99 |
| **Current or prior smoking exposure** | 4 (44.4) | 372 (50.5) | 0.75 |

Values are presented as absolute numbers (percentages) or median (interquartile range). Percentages are calculated per column. Differences between columns were assessed with a chi-square test or a Mann-Whitney U test as appropriate. DDR – DNA damage repair

**Supplementary table S3. Occurrence of VTE during follow-up stratified according to tumor type and CH carrier status.**

| **Tumor type** | **CH carrier status** | **VTE during follow-up (n=86)** | **No VTE during follow-up (n=881)** |
| --- | --- | --- | --- |
| **Brain** | CH (n=13) | 0 | 13 (100) |
|  | No CH (n=171) | 13 (7.6) | 158 (92.4) |
| **Breast** | CH (n=3) | 0 | 3 (100) |
|  | No CH (n=156) | 7 (4.5) | 149 (95.5) |
| **Lung** | CH (n=9) | 1 (11.1) | 8 (88.9) |
|  | No CH (n=194) | 21 (10.8) | 173 (90.2) |
| **Stomach** | CH (n=0) | 0 | 0 |
|  | No CH (n=15) | 1 (6.7) | 14 (93.3) |
| **Colorectal** | CH (n=2) | 1 (50.0) | 1 (50.0) |
|  | No CH (n=71) | 7 (9.9) | 64 (90.1) |
| **Pancreas** | CH (n=2) | 1 (50.0) | 1 (50.0) |
|  | No CH (n=71) | 13 (18.3) | 58 (81.7) |
| **Kidney** | CH (n=0) | 0 | 0 |
|  | No CH (n=9) | 1 (11.1) | 8 (88.9) |
| **Prostate** | CH (n=4) | 0 | 4 (100) |
|  | No CH (n=34) | 1 (2.9) | 33 (97.1) |
| **Multiple Myeloma** | CH (n=1) | 0 | 1 (100) |
|  | No CH (n=19) | 3 (15.8) | 16 (84.2) |
| **Lymphoma** | CH (n=4) | 0 | 4 (100) |
|  | No CH (n=95) | 2 (2.1) | 93 (97.9) |
| **Other** | CH (n=8) | 0 | 8 (100) |
|  | No CH (n=86) | 14 (16.3) | 72 (83.7) |

Values are presented as absolute numbers (percentages) or median (interquartile range). Percentages are calculated per row. CH – clonal hematopoiesis, VTE – venous thromboembolism

**Supplementary table S4. Occurrence of ATE during follow-up stratified according to tumor type and CH carrier status.**

| **Tumor type** | **CH carrier status** | **ATE during follow-up (n=18)** | **No ATE during follow-up (n=949)** |
| --- | --- | --- | --- |
| **Brain** | CH (n=13) | 0 | 13 (100) |
|  | No CH (n=171) | 4 (2.3) | 171 (97.7) |
| **Breast** | CH (n=3) | 0 | 3 (100) |
|  | No CH (n=156) | 0 | 156 (100) |
| **Lung** | CH (n=9) | 0 | 9 (100) |
|  | No CH (n=194) | 6 (3.1) | 194 (96.9) |
| **Stomach** | CH (n=0) | 0 | 0 |
|  | No CH (n=15) | 1 (6.7) | 14 (93.3) |
| **Colorectal** | CH (n=2) | 0 | 2 (100) |
|  | No CH (n=71) | 0 | 71 (100) |
| **Pancreas** | CH (n=2) | 0 | 2 (100) |
|  | No CH (n=71) | 0 | 71 (100) |
| **Kidney** | CH (n=0) | 0 | 0 |
|  | No CH (n=9) | 1 (11.1) | 8 (88.9) |
| **Prostate** | CH (n=4) | 0 | 4 (100) |
|  | No CH (n=34) | 2 (6.3) | 32 (93.7) |
| **Multiple Myeloma** | CH (n=1) | 0 | 1 (100) |
|  | No CH (n=19) | 0 | 19 (100) |
| **Lymphoma** | CH (n=4) | 0 | 4 (100) |
|  | No CH (n=95) | 2 (2.1) | 95 (97.9) |
| **Other** | CH (n=8) | 1 (12.5) | 7 (87.5) |
|  | No CH (n=86) | 1 (1.2) | 85 (98.8) |

Values are presented as absolute numbers (percentages) or median (interquartile range). Percentages are calculated per row. CH – clonal hematopoiesis, ATE – arterial thromboembolic events

**Supplementary table S5. Laboratory values and hemostatic biomarker levels in patients with and without DNA damage repair gene mutation.**

|  | **DDR gene mutation (n=11)** | **No DDR gene mutation (n=956)** | **p value** |
| --- | --- | --- | --- |
| **Hemoglobin (g/dL)** | 12.3 (10.5-13.2) | 13.1 (11.9-14.2) | 0.16 |
| **Platelets (10^9^/L)** | 236 (190-289) | 261 (208-321) | 0.25 |
| **Leucocytes (10^9^/L)** | 10.8 (7.6-15.0) | 7.6 (6.1-9.9) | 0.02 |
| **RDW (%)** | 15.1 (13.4-17.0) | 13.8 (13.1-14.6) | 0.002 |
| **CRP (mg/dL)** | 0.38 (0.17-0.47) | 0.42 (0.14-1.45) | 0.65 |
| **Fibrinogen (mg/dL)** | 338 (246-435) | 376 (308-467) | 0.23 |
| **Factor VIII activity (%)** | 273 (199-333) | 198 (153-250) | 0.02 |
| **D-dimer (µg/mL)** | 1.13 (0.64-1.71) | 0.73 (0.39-1.57) | 0.29 |
| **sP-selectin (ng/mL)** | 32.0 (26.8-32.5) | 34.6 (25.3-44.0) | 0.37 |
| **EV-TF (pg/mL)** | 0.17 (0.17-0.17) | 0.17 (0.05-0.28) | 0.99 |

Data presented as median (interquartile range). Differences between columns were assessed with a Mann-Whitney U Test. RDW – red cell distribution width, CRP – C-reactive protein, DDR – DNA damage repair

**Supplementary table S6. Laboratory values and hemostatic biomarker levels in patients with low thrombotic risk tumor types (n=197) with and without clonal hematopoiesis.**

|  | **CH (n=7)** | **No CH (n=190)** | **p value** |
| --- | --- | --- | --- |
| **Hemoglobin (g/dL)** | 12.9 (12.3-14.1) | 13.2 (12.5-14.1) | 0.34 |
| **Platelets (G/L)** | 226 (168-294) | 257 (215-307) | 0.35 |
| **Leucocytes (G/L)** | 7.5 (6.6-10.5) | 7.0 (6.0-8.7) | 0.34 |
| **RDW (%)** | 13.3 (12.6-14.8) | 13.3 (12.9-13.8) | 0.95 |
| **CRP (mg/dL)** | 0.28 (0.12-0.39) | 0.21 (0.11-0.46) | 0.91 |
| **Fibrinogen (mg/dL)** | 359 (332-412) | 347 (295-401) | 0.70 |
| **Factor VIII activity (%)** | 171 (159-276) | 175 (139-212) | 0.43 |
| **D-dimer (µg/mL)** | 0.61 (0.39-2.00) | 0.46 (0.28-0.87) | 0.33 |
| **sP-selectin (ng/mL)** | 32.0 (24.0-39.5) | 31.1 (22.6-41.7) | 0.81 |

Data presented as median (interquartile range). Differences between columns were assessed with a Mann-Whitney U Test. RDW – red cell distribution width, CRP – c-reactive protein, CH – clonal hematopoiesis

**Supplementary figure S1. Association of clonal hematopoiesis with all-cause mortality in uni- and multivariable analyses.**


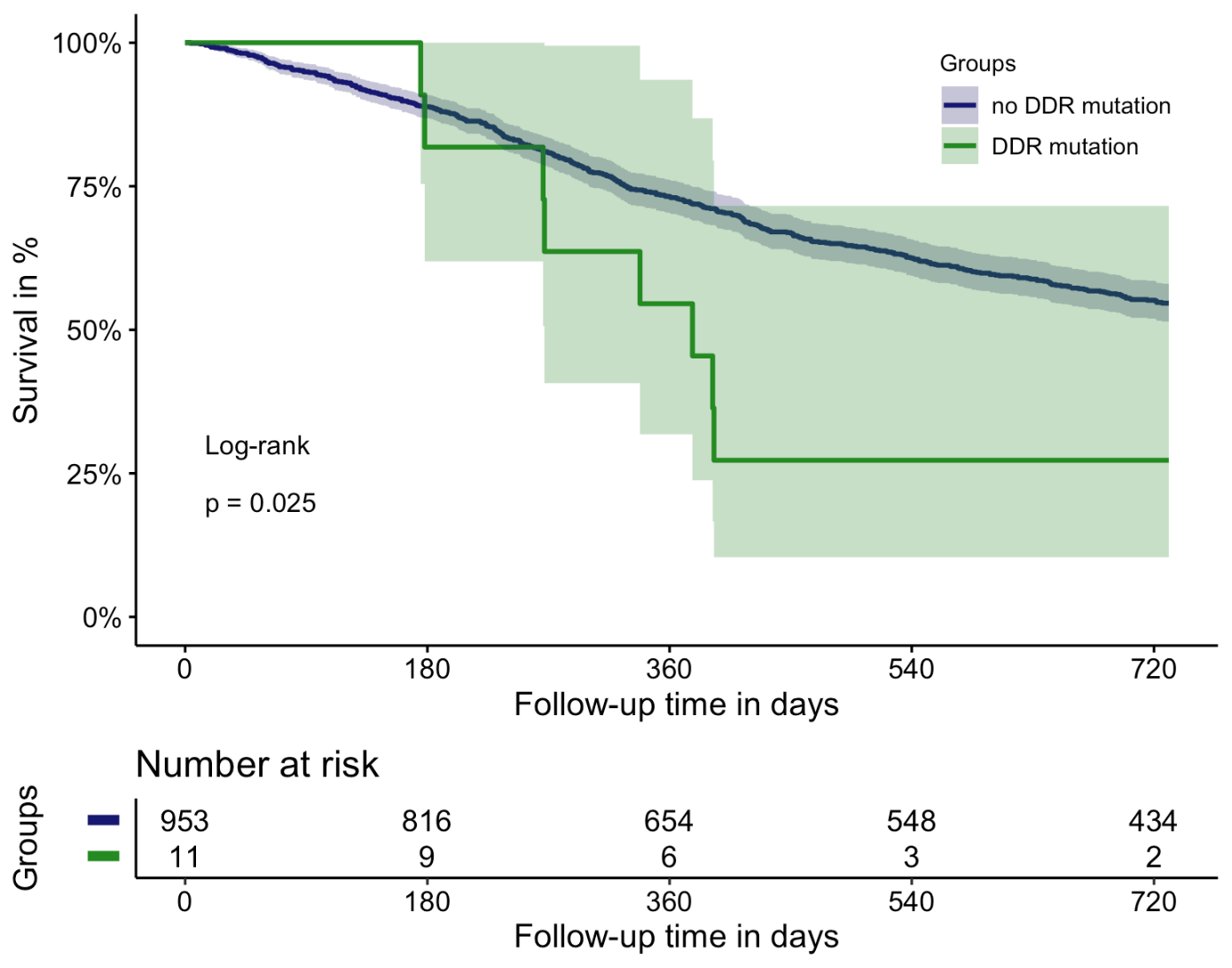


**Supplementary figure S2.** **Overall-survival in patients with (n=11) and without (n=956) DNA damage repair (DDR) gene mutation.** Patients were divided according to presence of a DDR gene mutation and the groups were compared within a Kaplan Meier analysis and with a log-rank test
